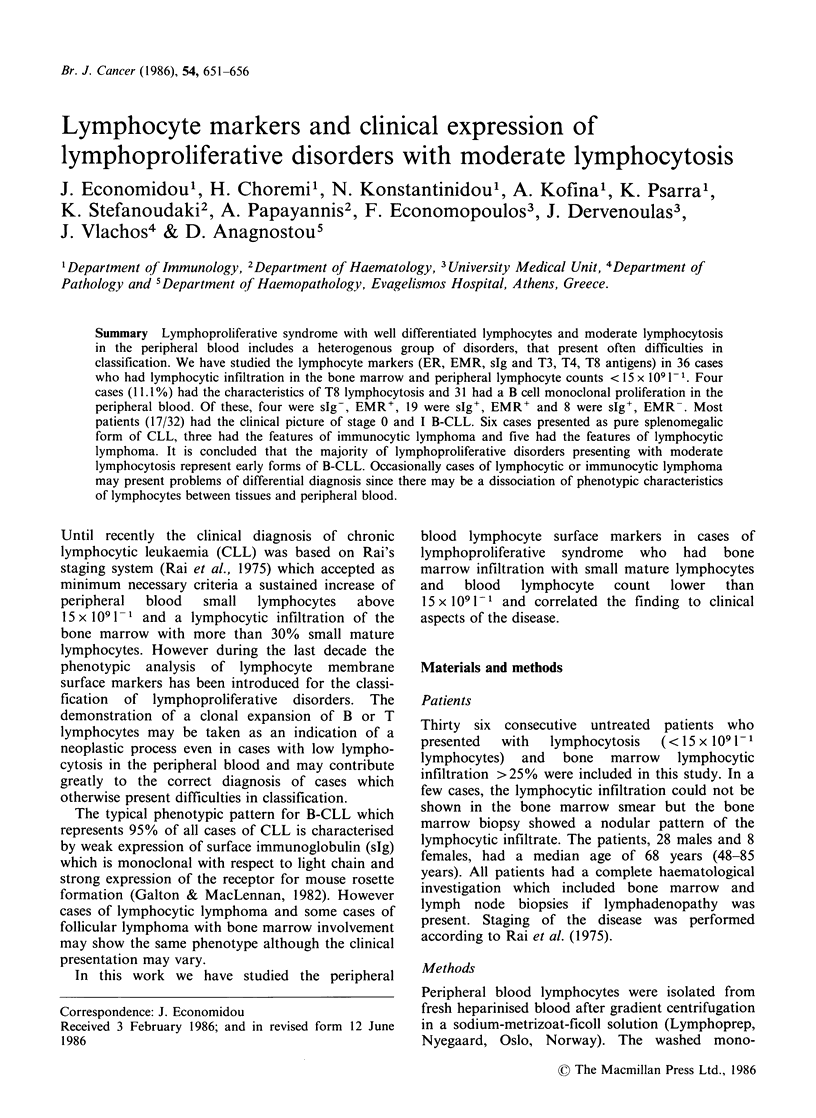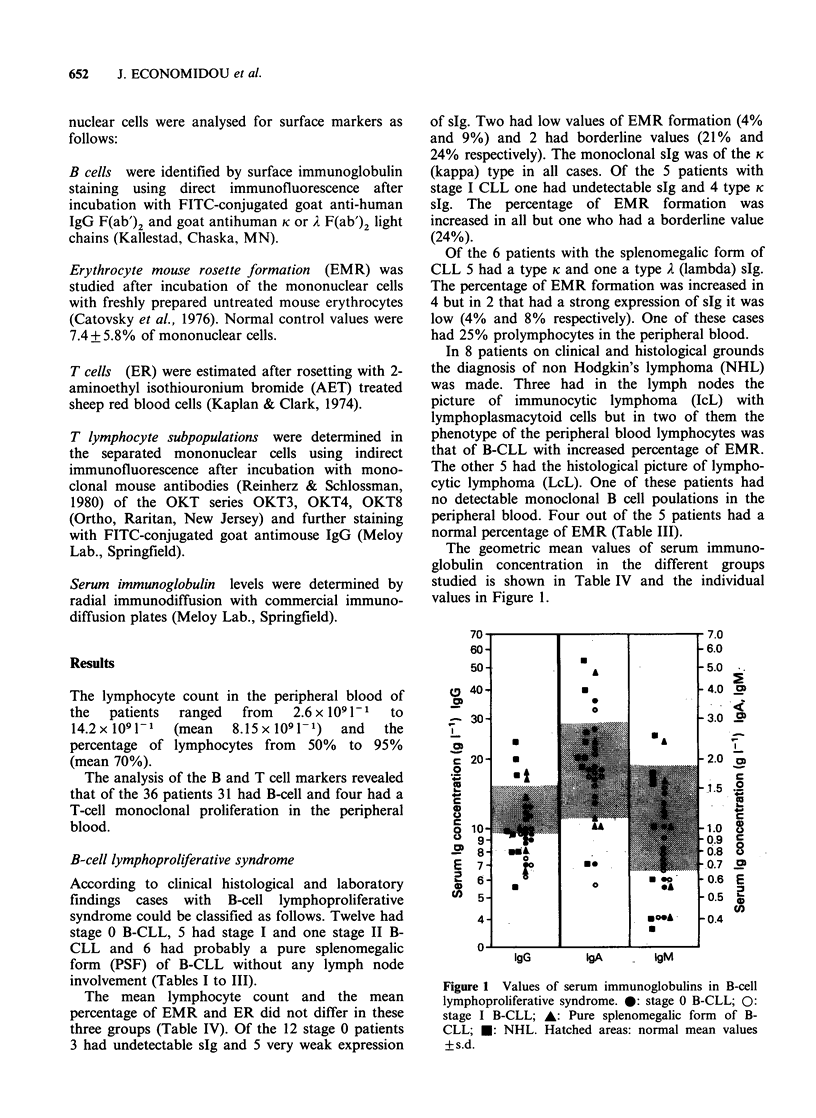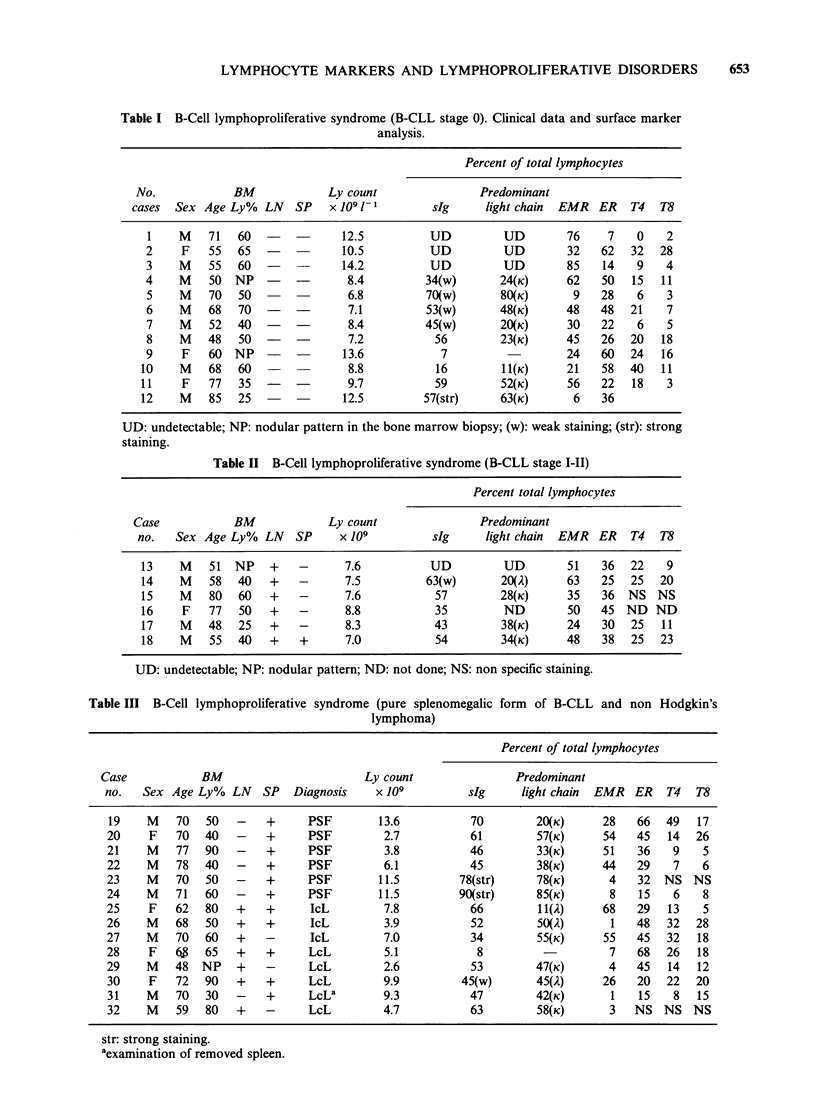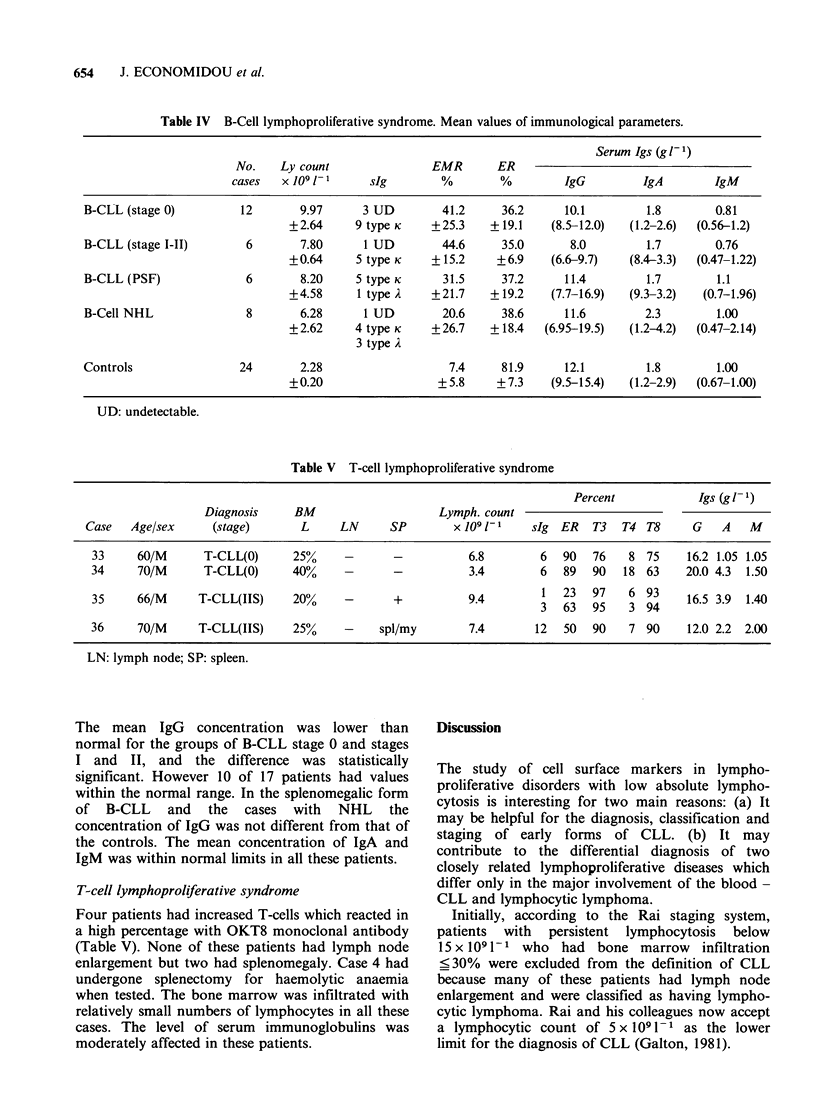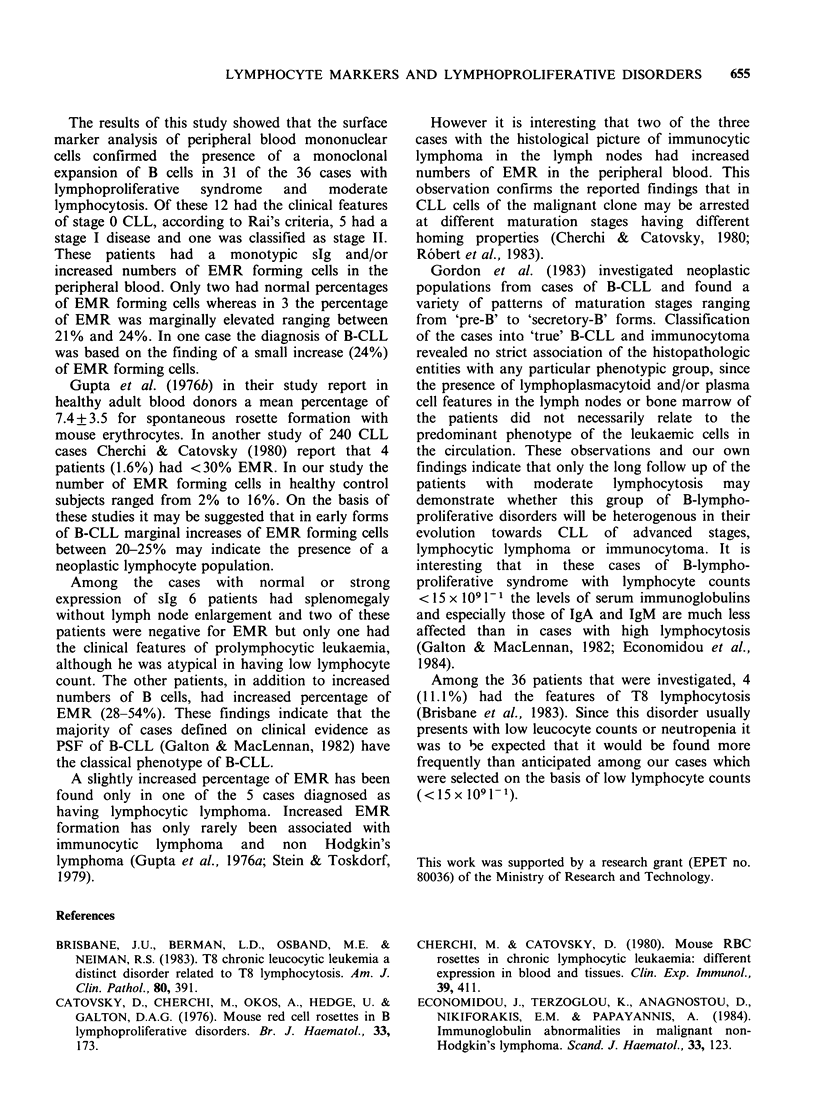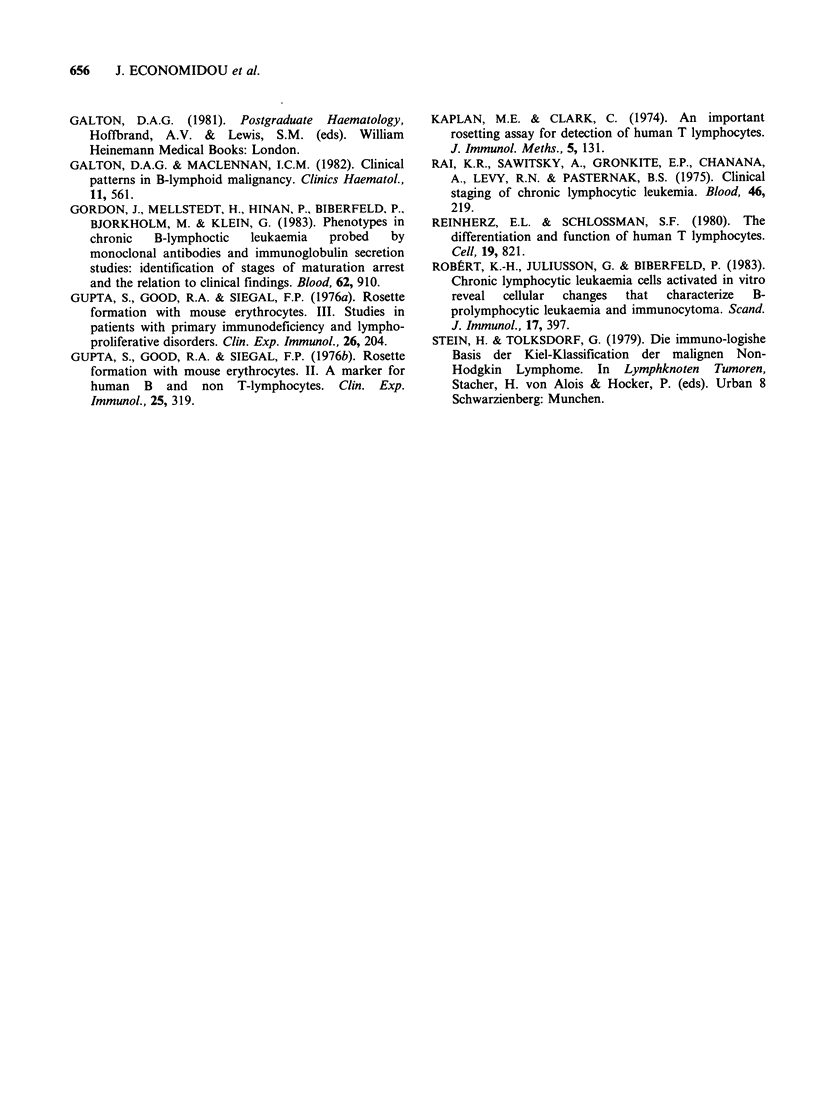# Lymphocyte markers and clinical expression of lymphoproliferative disorders with moderate lymphocytosis.

**DOI:** 10.1038/bjc.1986.222

**Published:** 1986-10

**Authors:** J. Economidou, H. Choremi, N. Konstantinidou, A. Kofina, K. Psarra, K. Stefanoudaki, A. Papayannis, F. Economopoulos, J. Dervenoulas, J. Vlachos

## Abstract

Lymphoproliferative syndrome with well differentiated lymphocytes and moderate lymphocytosis in the peripheral blood includes a heterogeneous group of disorders, that present often difficulties in classification. We have studied the lymphocyte markers (ER, EMR, sIg and T3, T4, T8 antigens) in 36 cases who had lymphocytic infiltration in the bone marrow and peripheral lymphocyte counts less than 15 X 10(9) l-1. Four cases (11.1%) had the characteristics of T8 lymphocytosis and 31 had a B cell monoclonal proliferation in the peripheral blood. Of these, four were sIg-, EMR+, 19 were sIg+, EMR+ and 8 were sIg+, EMR-. Most patients (17/32) had the clinical picture of stage 0 and I B-CLL. Six cases presented as pure splenomegalic form of CLL, three had the features of immunocytic lymphoma and five had the features of lymphocytic lymphoma. It is concluded that the majority of lymphoproliferative disorders presenting with moderate lymphocytosis represent early forms of B-CLL. Occasionally cases of lymphocytic or immunocytic lymphoma may present problems of differential diagnosis since there may be a dissociation of phenotypic characteristics of lymphocytes between tissues and peripheral blood.


Br. J. Cancer (1986), 54, 651–656

### Lymphocyte markers and clinical expression of lymphoproliferative disorders with moderate lymphocytosis

J. Economidou[1], H. Choremi[1], N. Konstantinidou[1], A. Kofina[1], K. Psarra[1], K. Stefanoudaki[2], A. Papayannis[2], F. Economopoulos[3], J. Dervenoulas[3], J. Vlachos[4] & D. Anagnostou[5]

[1]*Department of Immunology,* [2]*Department of Haematology,* [3]*University Medical Unit,* [4]*Department of Pathology and* [5]*Department of Haemopathology, Evagelismos Hospital, Athens, Greece.*

**Summary** Lymphoproliferative syndrome with well differentiated lymphocytes and moderate lymphocytosis in the peripheral blood includes a heterogenous group of disorders, that present often difficulties in classification. We have studied the lymphocyte markers (ER, EMR, sIg and T3, T4, T8 antigens) in 36 cases who had lymphocytic infiltration in the bone marrow and peripheral lymphocyte counts $<15\times10^9\,l^{-1}$. Four cases (11.1%) had the characteristics of T8 lymphocytosis and 31 had a B cell monoclonal proliferation in the peripheral blood. Of these, four were $sIg^-$, $EMR^+$, 19 were $sIg^+$, $EMR^+$ and 8 were $sIg^+$, $EMR^-$. Most patients (17/32) had the clinical picture of stage 0 and I B-CLL. Six cases presented as pure splenomegalic form of CLL, three had the features of immunocytic lymphoma and five had the features of lymphocytic lymphoma. It is concluded that the majority of lymphoproliferative disorders presenting with moderate lymphocytosis represent early forms of B-CLL. Occasionally cases of lymphocytic or immunocytic lymphoma may present problems of differential diagnosis since there may be a dissociation of phenotypic characteristics of lymphocytes between tissues and peripheral blood.

Until recently the clinical diagnosis of chronic lymphocytic leukaemia (CLL) was based on Rai's staging system (Rai *et al.*, 1975) which accepted as minimum necessary criteria a sustained increase of peripheral blood small lymphocytes above $15\times10^9\,l^{-1}$ and a lymphocytic infiltration of the bone marrow with more than 30% small mature lymphocytes. However during the last decade the phenotypic analysis of lymphocyte membrane surface markers has been introduced for the classification of lymphoproliferative disorders. The demonstration of a clonal expansion of B or T lymphocytes may be taken as an indication of a neoplastic process even in cases with low lymphocytosis in the peripheral blood and may contribute greatly to the correct diagnosis of cases which otherwise present difficulties in classification.

The typical phenotypic pattern for B-CLL which represents 95% of all cases of CLL is characterised by weak expression of surface immunoglobulin (sIg) which is monoclonal with respect to light chain and strong expression of the receptor for mouse rosette formation (Galton & MacLennan, 1982). However cases of lymphocytic lymphoma and some cases of follicular lymphoma with bone marrow involvement may show the same phenotype although the clinical presentation may vary.

In this work we have studied the peripheral blood lymphocyte surface markers in cases of lymphoproliferative syndrome who had bone marrow infiltration with small mature lymphocytes and blood lymphocyte count lower than $15\times10^9\,l^{-1}$ and correlated the finding to clinical aspects of the disease.

Correspondence: J. Economidou
Received 3 February 1986; and in revised form 12 June 1986

### Materials and methods

### *Patients*

Thirty six consecutive untreated patients who presented with lymphocytosis ($<15\times10^9\,l^{-1}$ lymphocytes) and bone marrow lymphocytic infiltration >25% were included in this study. In a few cases, the lymphocytic infiltration could not be shown in the bone marrow smear but the bone marrow biopsy showed a nodular pattern of the lymphocytic infiltrate. The patients, 28 males and 8 females, had a median age of 68 years (48–85 years). All patients had a complete haematological investigation which included bone marrow and lymph node biopsies if lymphadenopathy was present. Staging of the disease was performed according to Rai *et al.* (1975).

### *Methods*

Peripheral blood lymphocytes were isolated from fresh heparinised blood after gradient centrifugation in a sodium-metrizoat-ficoll solution (Lymphoprep, Nyegaard, Oslo, Norway). The washed mono-

© The Macmillan Press Ltd., 1986

nuclear cells were analysed for surface markers as follows:

*B cells* were identified by surface immunoglobulin staining using direct immunofluorescence after incubation with FITC-conjugated goat anti-human IgG $F(ab')_2$ and goat antihuman $\kappa$ or $\lambda$ $F(ab')_2$ light chains (Kallestad, Chaska, MN).

*Erythrocyte mouse rosette formation* (EMR) was studied after incubation of the mononuclear cells with freshly prepared untreated mouse erythrocytes (Catovsky *et al.*, 1976). Normal control values were $7.4 \pm 5.8\%$ of mononuclear cells.

*T cells* (ER) were estimated after rosetting with 2-aminoethyl isothiouronium bromide (AET) treated sheep red blood cells (Kaplan & Clark, 1974).

*T lymphocyte subpopulations* were determined in the separated mononuclear cells using indirect immunofluorescence after incubation with monoclonal mouse antibodies (Reinherz & Schlossman, 1980) of the OKT series OKT3, OKT4, OKT8 (Ortho, Raritan, New Jersey) and further staining with FITC-conjugated goat antimouse IgG (Meloy Lab., Springfield).

*Serum immunoglobulin* levels were determined by radial immunodiffusion with commercial immunodiffusion plates (Meloy Lab., Springfield).

### Results

The lymphocyte count in the peripheral blood of the patients ranged from $2.6 \times 10^9 \, l^{-1}$ to $14.2 \times 10^9 \, l^{-1}$ (mean $8.15 \times 10^9 \, l^{-1}$) and the percentage of lymphocytes from 50% to 95% (mean 70%).

The analysis of the B and T cell markers revealed that of the 36 patients 31 had B-cell and four had a T-cell monoclonal proliferation in the peripheral blood.

### *B-cell lymphoproliferative syndrome*

According to clinical histological and laboratory findings cases with B-cell lymphoproliferative syndrome could be classified as follows. Twelve had stage 0 B-CLL, 5 had stage I and one stage II B-CLL and 6 had probably a pure splenomegalic form (PSF) of B-CLL without any lymph node involvement (Tables I to III).

The mean lymphocyte count and the mean percentage of EMR and ER did not differ in these three groups (Table IV). Of the 12 stage 0 patients 3 had undetectable sIg and 5 very weak expression of sIg. Two had low values of EMR formation (4% and 9%) and 2 had borderline values (21% and 24% respectively). The monoclonal sIg was of the $\kappa$ (kappa) type in all cases. Of the 5 patients with stage I CLL one had undetectable sIg and 4 type $\kappa$ sIg. The percentage of EMR formation was increased in all but one who had a borderline value (24%).

Of the 6 patients with the splenomegalic form of CLL 5 had a type $\kappa$ and one a type $\lambda$ (lambda) sIg. The percentage of EMR formation was increased in 4 but in 2 that had a strong expression of sIg it was low (4% and 8% respectively). One of these cases had 25% prolymphocytes in the peripheral blood.

In 8 patients on clinical and histological grounds the diagnosis of non Hodgkin's lymphoma (NHL) was made. Three had in the lymph nodes the picture of immunocytic lymphoma (IcL) with lymphoplasmacytoid cells but in two of them the phenotype of the peripheral blood lymphocytes was that of B-CLL with increased percentage of EMR. The other 5 had the histological picture of lymphocytic lymphoma (LcL). One of these patients had no detectable monoclonal B cell poulations in the peripheral blood. Four out of the 5 patients had a normal percentage of EMR (Table III).

The geometric mean values of serum immunoglobulin concentration in the different groups studied is shown in Table IV and the individual values in Figure 1.

**Figure 1** Values of serum immunoglobulins in B-cell lymphoproliferative syndrome. ●: stage 0 B-CLL; ○: stage I B-CLL; ▲: Pure splenomegalic form of B-CLL; ■: NHL. Hatched areas: normal mean values ±s.d.

**Table I** B-Cell lymphoproliferative syndrome (B-CLL stage 0). Clinical data and surface marker analysis.

| | | | | | | | *Percent of total lymphocytes* | | | | | |
|---|---|---|---|---|---|---|---|---|---|---|---|---|
| *No. cases* | *Sex* | *Age* | *BM Ly%* | *LN* | *SP* | *Ly count* $\times 10^9 l^{-1}$ | *sIg* | *Predominant light chain* | *EMR* | *ER* | *T4* | *T8* |
| 1 | M | 71 | 60 | — | — | 12.5 | UD | UD | 76 | 7 | 0 | 2 |
| 2 | F | 55 | 65 | — | — | 10.5 | UD | UD | 32 | 62 | 32 | 28 |
| 3 | M | 55 | 60 | — | — | 14.2 | UD | UD | 85 | 14 | 9 | 4 |
| 4 | M | 50 | NP | — | — | 8.4 | 34(w) | 24($\kappa$) | 62 | 50 | 15 | 11 |
| 5 | M | 70 | 50 | — | — | 6.8 | 70(w) | 80($\kappa$) | 9 | 28 | 6 | 3 |
| 6 | M | 68 | 70 | — | — | 7.1 | 53(w) | 48($\kappa$) | 48 | 48 | 21 | 7 |
| 7 | M | 52 | 40 | — | — | 8.4 | 45(w) | 20($\kappa$) | 30 | 22 | 6 | 5 |
| 8 | M | 48 | 50 | — | — | 7.2 | 56 | 23($\kappa$) | 45 | 26 | 20 | 18 |
| 9 | F | 60 | NP | — | — | 13.6 | 7 | — | 24 | 60 | 24 | 16 |
| 10 | M | 68 | 60 | — | — | 8.8 | 16 | 11($\kappa$) | 21 | 58 | 40 | 11 |
| 11 | F | 77 | 35 | — | — | 9.7 | 59 | 52($\kappa$) | 56 | 22 | 18 | 3 |
| 12 | M | 85 | 25 | — | — | 12.5 | 57(str) | 63($\kappa$) | 6 | 36 | | |

UD: undetectable; NP: nodular pattern in the bone marrow biopsy; (w): weak staining; (str): strong staining.

**Table II** B-Cell lymphoproliferative syndrome (B-CLL stage I-II)

| | | | | | | | *Percent total lymphocytes* | | | | | |
|---|---|---|---|---|---|---|---|---|---|---|---|---|
| *Case no.* | *Sex* | *Age* | *BM Ly%* | *LN* | *SP* | *Ly count* $\times 10^9$ | *sIg* | *Predominant light chain* | *EMR* | *ER* | *T4* | *T8* |
| 13 | M | 51 | NP | + | – | 7.6 | UD | UD | 51 | 36 | 22 | 9 |
| 14 | M | 58 | 40 | + | – | 7.5 | 63(w) | 20($\lambda$) | 63 | 25 | 25 | 20 |
| 15 | M | 80 | 60 | + | – | 7.6 | 57 | 28($\kappa$) | 35 | 36 | NS | NS |
| 16 | F | 77 | 50 | + | – | 8.8 | 35 | ND | 50 | 45 | ND | ND |
| 17 | M | 48 | 25 | + | – | 8.3 | 43 | 38($\kappa$) | 24 | 30 | 25 | 11 |
| 18 | M | 55 | 40 | + | + | 7.0 | 54 | 34($\kappa$) | 48 | 38 | 25 | 23 |

UD: undetectable; NP: nodular pattern; ND: not done; NS: non specific staining.

**Table III** B-Cell lymphoproliferative syndrome (pure splenomegalic form of B-CLL and non Hodgkin's lymphoma)

| | | | | | | | | *Percent of total lymphocytes* | | | | | |
|---|---|---|---|---|---|---|---|---|---|---|---|---|---|
| *Case no.* | *Sex* | *Age* | *BM Ly%* | *LN* | *SP* | *Diagnosis* | *Ly count* $\times 10^9$ | *sIg* | *Predominant light chain* | *EMR* | *ER* | *T4* | *T8* |
| 19 | M | 70 | 50 | – | + | PSF | 13.6 | 70 | 20($\kappa$) | 28 | 66 | 49 | 17 |
| 20 | F | 70 | 40 | – | + | PSF | 2.7 | 61 | 57($\kappa$) | 54 | 45 | 14 | 26 |
| 21 | M | 77 | 90 | – | + | PSF | 3.8 | 46 | 33($\kappa$) | 51 | 36 | 9 | 5 |
| 22 | M | 78 | 40 | – | + | PSF | 6.1 | 45 | 38($\kappa$) | 44 | 29 | 7 | 6 |
| 23 | M | 70 | 50 | – | + | PSF | 11.5 | 78(str) | 78($\kappa$) | 4 | 32 | NS | NS |
| 24 | M | 71 | 60 | – | + | PSF | 11.5 | 90(str) | 85($\kappa$) | 8 | 15 | 6 | 8 |
| 25 | F | 62 | 80 | + | + | IcL | 7.8 | 66 | 11($\lambda$) | 68 | 29 | 13 | 5 |
| 26 | M | 68 | 50 | + | + | IcL | 3.9 | 52 | 50($\lambda$) | 1 | 48 | 32 | 28 |
| 27 | M | 70 | 60 | + | – | IcL | 7.0 | 34 | 55($\kappa$) | 55 | 45 | 32 | 18 |
| 28 | F | 68 | 65 | + | + | LcL | 5.1 | 8 | — | 7 | 68 | 26 | 18 |
| 29 | M | 48 | NP | + | – | LcL | 2.6 | 53 | 47($\kappa$) | 4 | 45 | 14 | 12 |
| 30 | F | 72 | 90 | + | + | LcL | 9.9 | 45(w) | 45($\lambda$) | 26 | 20 | 22 | 20 |
| 31 | M | 70 | 30 | – | + | LcL[a] | 9.3 | 47 | 42($\kappa$) | 1 | 15 | 8 | 15 |
| 32 | M | 59 | 80 | + | – | LcL | 4.7 | 63 | 58($\kappa$) | 3 | NS | NS | NS |

str: strong staining.
[a]examination of removed spleen.

**Table IV** B-Cell lymphoproliferative syndrome. Mean values of immunological parameters.

| | *No. cases* | *Ly count* $\times 10^9 l^{-1}$ | *sIg* | *EMR* % | *ER* % | *Serum Igs* ($g l^{-1}$) *IgG* | *IgA* | *IgM* |
|---|---|---|---|---|---|---|---|---|
| B-CLL (stage 0) | 12 | 9.97<br>±2.64 | 3 UD<br>9 type κ | 41.2<br>±25.3 | 36.2<br>±19.1 | 10.1<br>(8.5–12.0) | 1.8<br>(1.2–2.6) | 0.81<br>(0.56–1.2) |
| B-CLL (stage I-II) | 6 | 7.80<br>±0.64 | 1 UD<br>5 type κ | 44.6<br>±15.2 | 35.0<br>±6.9 | 8.0<br>(6.6–9.7) | 1.7<br>(8.4–3.3) | 0.76<br>(0.47–1.22) |
| B-CLL (PSF) | 6 | 8.20<br>±4.58 | 5 type κ<br>1 type λ | 31.5<br>±21.7 | 37.2<br>±19.2 | 11.4<br>(7.7–16.9) | 1.7<br>(9.3–3.2) | 1.1<br>(0.7–1.96) |
| B-Cell NHL | 8 | 6.28<br>±2.62 | 1 UD<br>4 type κ<br>3 type λ | 20.6<br>±26.7 | 38.6<br>±18.4 | 11.6<br>(6.95–19.5) | 2.3<br>(1.2–4.2) | 1.00<br>(0.47–2.14) |
| Controls | 24 | 2.28<br>±0.20 | | 7.4<br>±5.8 | 81.9<br>±7.3 | 12.1<br>(9.5–15.4) | 1.8<br>(1.2–2.9) | 1.00<br>(0.67–1.00) |

UD: undetectable.

**Table V** T-cell lymphoproliferative syndrome

| *Case* | *Age/sex* | *Diagnosis (stage)* | *BM L* | *LN* | *SP* | *Lymph. count* $\times 10^9 l^{-1}$ | *Percent* *sIg* | *ER* | *T3* | *T4* | *T8* | *Igs* ($g l^{-1}$) *G* | *A* | *M* |
|---|---|---|---|---|---|---|---|---|---|---|---|---|---|---|
| 33 | 60/M | T-CLL(0) | 25% | – | – | 6.8 | 6 | 90 | 76 | 8 | 75 | 16.2 | 1.05 | 1.05 |
| 34 | 70/M | T-CLL(0) | 40% | – | – | 3.4 | 6 | 89 | 90 | 18 | 63 | 20.0 | 4.3 | 1.50 |
| 35 | 66/M | T-CLL(IIS) | 20% | – | + | 9.4 | 1<br>3 | 23<br>63 | 97<br>95 | 6<br>3 | 93<br>94 | 16.5 | 3.9 | 1.40 |
| 36 | 70/M | T-CLL(IIS) | 25% | – | spl/my | 7.4 | 12 | 50 | 90 | 7 | 90 | 12.0 | 2.2 | 2.00 |

LN: lymph node; SP: spleen.

The mean IgG concentration was lower than normal for the groups of B-CLL stage 0 and stages I and II, and the difference was statistically significant. However 10 of 17 patients had values within the normal range. In the splenomegalic form of B-CLL and the cases with NHL the concentration of IgG was not different from that of the controls. The mean concentration of IgA and IgM was within normal limits in all these patients.

### *T-cell lymphoproliferative syndrome*

Four patients had increased T-cells which reacted in a high percentage with OKT8 monoclonal antibody (Table V). None of these patients had lymph node enlargement but two had splenomegaly. Case 4 had undergone splenectomy for haemolytic anaemia when tested. The bone marrow was infiltrated with relatively small numbers of lymphocytes in all these cases. The level of serum immunoglobulins was moderately affected in these patients.

### Discussion

The study of cell surface markers in lymphoproliferative disorders with low absolute lymphocytosis is interesting for two main reasons: (a) It may be helpful for the diagnosis, classification and staging of early forms of CLL. (b) It may contribute to the differential diagnosis of two closely related lymphoproliferative diseases which differ only in the major involvement of the blood – CLL and lymphocytic lymphoma.

Initially, according to the Rai staging system, patients with persistent lymphocytosis below $15 \times 10^9 l^{-1}$ who had bone marrow infiltration $\leq 30\%$ were excluded from the definition of CLL because many of these patients had lymph node enlargement and were classified as having lymphocytic lymphoma. Rai and his colleagues now accept a lymphocytic count of $5 \times 10^9 l^{-1}$ as the lower limit for the diagnosis of CLL (Galton, 1981).

The results of this study showed that the surface marker analysis of peripheral blood mononuclear cells confirmed the presence of a monoclonal expansion of B cells in 31 of the 36 cases with lymphoproliferative syndrome and moderate lymphocytosis. Of these 12 had the clinical features of stage 0 CLL, according to Rai's criteria, 5 had a stage I disease and one was classified as stage II. These patients had a monotypic sIg and/or increased numbers of EMR forming cells in the peripheral blood. Only two had normal percentages of EMR forming cells whereas in 3 the percentage of EMR was marginally elevated ranging between 21% and 24%. In one case the diagnosis of B-CLL was based on the finding of a small increase (24%) of EMR forming cells.

Gupta *et al.* (1976*b*) in their study report in healthy adult blood donors a mean percentage of $7.4 \pm 3.5$ for spontaneous rosette formation with mouse erythrocytes. In another study of 240 CLL cases Cherchi & Catovsky (1980) report that 4 patients (1.6%) had <30% EMR. In our study the number of EMR forming cells in healthy control subjects ranged from 2% to 16%. On the basis of these studies it may be suggested that in early forms of B-CLL marginal increases of EMR forming cells between 20–25% may indicate the presence of a neoplastic lymphocyte population.

Among the cases with normal or strong expression of sIg 6 patients had splenomegaly without lymph node enlargement and two of these patients were negative for EMR but only one had the clinical features of prolymphocytic leukaemia, although he was atypical in having low lymphocyte count. The other patients, in addition to increased numbers of B cells, had increased percentage of EMR (28–54%). These findings indicate that the majority of cases defined on clinical evidence as PSF of B-CLL (Galton & MacLennan, 1982) have the classical phenotype of B-CLL.

A slightly increased percentage of EMR has been found only in one of the 5 cases diagnosed as having lymphocytic lymphoma. Increased EMR formation has only rarely been associated with immunocytic lymphoma and non Hodgkin's lymphoma (Gupta *et al.*, 1976*a*; Stein & Toskdorf, 1979).

However it is interesting that two of the three cases with the histological picture of immunocytic lymphoma in the lymph nodes had increased numbers of EMR in the peripheral blood. This observation confirms the reported findings that in CLL cells of the malignant clone may be arrested at different maturation stages having different homing properties (Cherchi & Catovsky, 1980; Róbert *et al.*, 1983).

Gordon *et al.* (1983) investigated neoplastic populations from cases of B-CLL and found a variety of patterns of maturation stages ranging from 'pre-B' to 'secretory-B' forms. Classification of the cases into 'true' B-CLL and immunocytoma revealed no strict association of the histopathologic entities with any particular phenotypic group, since the presence of lymphoplasmacytoid and/or plasma cell features in the lymph nodes or bone marrow of the patients did not necessarily relate to the predominant phenotype of the leukaemic cells in the circulation. These observations and our own findings indicate that only the long follow up of the patients with moderate lymphocytosis may demonstrate whether this group of B-lymphoproliferative disorders will be heterogenous in their evolution towards CLL of advanced stages, lymphocytic lymphoma or immunocytoma. It is interesting that in these cases of B-lymphoproliferative syndrome with lymphocyte counts $<15 \times 10^9 l^{-1}$ the levels of serum immunoglobulins and especially those of IgA and IgM are much less affected than in cases with high lymphocytosis (Galton & MacLennan, 1982; Economidou *et al.*, 1984).

Among the 36 patients that were investigated, 4 (11.1%) had the features of T8 lymphocytosis (Brisbane *et al.*, 1983). Since this disorder usually presents with low leucocyte counts or neutropenia it was to be expected that it would be found more frequently than anticipated among our cases which were selected on the basis of low lymphocyte counts ($<15 \times 10^9 l^{-1}$).

This work was supported by a research grant (EPET no. 80036) of the Ministry of Research and Technology.

### References

BRISBANE, J.U., BERMAN, L.D., OSBAND, M.E. & NEIMAN, R.S. (1983). T8 chronic leucocytic leukemia a distinct disorder related to T8 lymphocytosis. *Am. J. Clin. Pathol.*, **80**, 391.

CATOVSKY, D., CHERCHI, M., OKOS, A., HEDGE, U. & GALTON, D.A.G. (1976). Mouse red cell rosettes in B lymphoproliferative disorders. *Br. J. Haematol.*, **33**, 173.

CHERCHI, M. & CATOVSKY, D. (1980). Mouse RBC rosettes in chronic lymphocytic leukaemia: different expression in blood and tissues. *Clin. Exp. Immunol.*, **39**, 411.

ECONOMIDOU, J., TERZOGLOU, K., ANAGNOSTOU, D., NIKIFORAKIS, E.M. & PAPAYANNIS, A. (1984). Immunoglobulin abnormalities in malignant non-Hodgkin's lymphoma. *Scand. J. Haematol.*, **33**, 123.

GALTON, D.A.G. (1981). *Postgraduate Haematology*, Hoffbrand, A.V. & Lewis, S.M. (eds). William Heinemann Medical Books: London.

GALTON, D.A.G. & MACLENNAN, I.C.M. (1982). Clinical patterns in B-lymphoid malignancy. *Clinics Haematol.*, **11**, 561.

GORDON, J., MELLSTEDT, H., HINAN, P., BIBERFELD, P., BJORKHOLM, M. & KLEIN, G. (1983). Phenotypes in chronic B-lymphoctic leukaemia probed by monoclonal antibodies and immunoglobulin secretion studies: identification of stages of maturation arrest and the relation to clinical findings. *Blood*, **62**, 910.

GUPTA, S., GOOD, R.A. & SIEGAL, F.P. (1976*a*). Rosette formation with mouse erythrocytes. III. Studies in patients with primary immunodeficiency and lymphoproliferative disorders. *Clin. Exp. Immunol.*, **26**, 204.

GUPTA, S., GOOD, R.A. & SIEGAL, F.P. (1976*b*). Rosette formation with mouse erythrocytes. II. A marker for human B and non T-lymphocytes. *Clin. Exp. Immunol.*, **25**, 319.

KAPLAN, M.E. & CLARK, C. (1974). An important rosetting assay for detection of human T lymphocytes. *J. Immunol. Meths.*, **5**, 131.

RAI, K.R., SAWITSKY, A., GRONKITE, E.P., CHANANA, A., LEVY, R.N. & PASTERNAK, B.S. (1975). Clinical staging of chronic lymphocytic leukemia. *Blood*, **46**, 219.

REINHERZ, E.L. & SCHLOSSMAN, S.F. (1980). The differentiation and function of human T lymphocytes. *Cell*, **19**, 821.

ROBÉRT, K.-H., JULIUSSON, G. & BIBERFELD, P. (1983). Chronic lymphocytic leukaemia cells activated in vitro reveal cellular changes that characterize B-prolymphocytic leukaemia and immunocytoma. *Scand. J. Immunol.*, **17**, 397.

STEIN, H. & TOLKSDORF, G. (1979). Die immuno-logishe Basis der Kiel-Klassification der malignen Non-Hodgkin Lymphome. In *Lymphknoten Tumoren*, Stacher, H. von Alois & Hocker, P. (eds). Urban 8 Schwarzienberg: Munchen.